# Dissecting the ecosystem service of large-scale pollutant retention: The role of wetlands and other landscape features

**DOI:** 10.1007/s13280-014-0594-8

**Published:** 2015-01-09

**Authors:** Andrew Quin, Fernando Jaramillo, Georgia Destouni

**Affiliations:** Department of Physical Geography and Quaternary Geology, Stockholm University, 106 91 Stockholm, Sweden

**Keywords:** Ecosystem service, Water quality, Wetlands, Pollutant retention, Landscape-scale, Baltic Sea eutrophication

## Abstract

Various features of a landscape contribute to the regulating ecosystem service of reducing waterborne pollutant loading to downstream environments. At local scales, wetlands have been shown to be effective in retaining pollutants. Here, we investigate the landscape-scale contribution to pollutant retention provided by multiple wetlands. We develop a general analytical model which shows that the retention contribution of wetlands and other landscape features is only significant if a large fraction of the total waterborne pollutant transport passes through them. Next, by means of a statistical analysis of official data, we quantify the nutrient retention contribution of wetlands for multiple sub-catchments in two Swedish Water Management Districts. We compare this with the retention contribution of two other landscape features: the waterborne transport distance and major lakes. The landscape-scale retention contribution of wetlands is undetectable; rather, the other two landscape features account for much of the total nutrient retention.

**Electronic supplementary material:**

The online version of this article (doi:10.1007/s13280-014-0594-8) contains supplementary material, which is available to authorized users.

## Introduction

Waterborne loads of pollutants through the landscape pose major threats to inland and coastal-marine water quality, ecosystems, water security, and health (Jarsjö et al. 2005; Darracq et al. 2005; Törnqvist et al. 2011; Nilsson et al. 2013). However, the landscape, including its surface water systems and subsurface pathways, provides a regulating ecosystem service: pollution control (Millennium Ecosystem Assessment 2005), which reduces pollutant loading from various sources to downstream water systems (Darracq et al. 2008; Destouni et al. 2010; Cvetkovic et al. 2012). Without this landscape-scale ecosystem service, pollutant loading through and from the landscape would be greater (Persson and Destouni 2009, 2011; Destouni et al. 2010) and would require more, and more costly, abatement measures in order to achieve the same level of protection for downstream waters (Baresel et al. 2006; Destouni et al. 2006).

The regulating ecosystem service of pollutant retention in the landscape is also closely linked to—and may even be critical for—other types of ecosystem services, such as clean water provision, nutrient cycling, and recreational water environments (Millennium Ecosystem Assessment 2005). Thus, the value of each of these other ecosystem services depends, to some degree, on the landscape-scale service of pollutant retention. New, parallel studies call for increased attention to trade-offs and synergisms among different ecosystem services (Queiroz et al. 2015), scale issues in the quantity and valuation of ecosystems services (Andersson et al. 2015) and improved theoretical understanding of multiple and non-linear interactions among ecosystem services in order to improve our ability to co-manage such services (Kremen and Ostfeld 2005; Tallis and Kareiva 2006; Carpenter et al. 2006a, b).

In particular, wetlands are often managed within a range of possibly conflicting and/or synergistic human requirements for ecosystem services, such as agricultural activities and conservation imperatives (Kininmonth et al. 2015); other ecosystem services with vegetation as their major biotic determinant (Moor et al. 2015); and pollutant reduction efforts for both point (e.g., wastewater treatment plants and industry) and diffuse (e.g., agriculture, forestry, unconnected wastewater pipes, storm water, and atmospheric deposition) sources (Gren et al. 2000; Mitsch et al. 2005; Baresel et al. 2006; Land et al. 2013).

Regarding pollutant reduction by wetlands, Reddy et al. (1999) have reviewed phosphorus retention in streams and wetlands; Vymazal (2007) has reviewed nutrient removal in various types of constructed wetlands; and Saunders and Kalff (2001) have compared the magnitude of nutrient retention in wetlands, lakes, and rivers, finding that wetlands retained the greatest amounts followed by lakes and then rivers. Other studies have also assessed how effective individual wetlands are in retaining pollutants that are carried by the water flow through them (Aisling et al. 1999; ERMITE-Consortium 2004; Mitsch et al. 2005). Among studies that have discussed wetland function in large-scale landscape contexts, Zedler et al. (2003) addressed the challenge of restoring vegetation on tidal, hypersaline substrates; Thiere et al. (2009) addressed the biodiversity benefits on local and regional scales of wetland creation in agricultural landscapes; and Tilley and Brown (2006) addressed the benefits of subtropical wetland stormwater management systems. Regarding the pollution reduction benefits of wetlands in large-scale contexts, the review paper by Zedler (2003) discussed this as a possible part of an overall wetland restoration strategy using an adaptive management approach. Also, the more recent review paper by Kröger et al. (2013) discussed it as a part of an overall approach to phosphorus management in agricultural landscapes.

There is thus evidence that wetlands can reduce pollutant loads carried by the water flowing through them, and there is an ongoing discussion of pollution reduction as a possible significant part of multiple ecosystem services that wetlands can provide on landscape scales. However, there still remains a need to quantitatively investigate how the evidenced wetland retention of waterborne pollutants passing through them actually functions as an overall regulating ecosystem service on large landscape and catchment scales. A scale-related ecosystem-service question for further investigation and quantification can then be formulated as what is the large-scale ecosystem service of pollutant retention by multiple wetlands distributed throughout the landscape?

This study tackles this question by breaking it down to (i) what is the large-scale contribution of wetlands to total pollutant retention in the landscape and (ii) how does this contribution compare with that of other landscape features? To answer these questions, a general analytical model is developed for the regulating ecosystem service of pollutant retention in the landscape. From this, a general condition is identified for wetlands and other landscape features to significantly affect landscape-scale pollutant retention. Whether or not this general condition is fulfilled at a landscape-scale is further assessed by means of a statistical analysis using official Swedish data. These data include wetland occurrence and nutrient catchment inputs and coastal outputs for multiple sub-catchments across two major Swedish Water Management Districts (WMDs): the North and the South Baltic WMDs. The landscape-scale nutrient retention effects of (a) wetlands are evaluated and compared with the effects of two other landscape features: (b) major lakes and (c) the transport distance along the surface flow network from each catchment outlet to the associated downstream coastal output.

## General analytical model

**Fig. 1:**
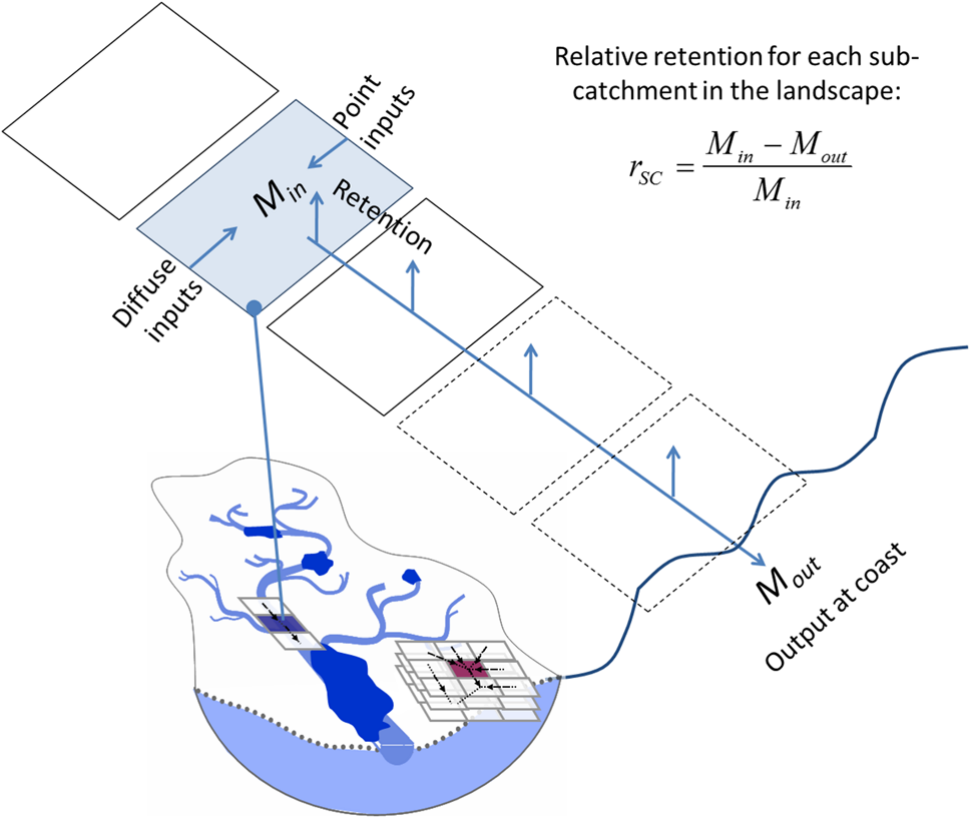
Schematic representation of the general conceptualization framework for pollutant retention in the landscape, showing relative retention *r*_SC_ for a sub-catchment. *M*_in_ is the total pollutant load to the sub-catchment from all point and diffuse source. *M*_out_ is the pollutant load which reaches the coastal outlet via the transport pathway after retention in the landscape

The relative retention (*r*_SC_, Fig. 1) of pollutant inputs from all (diffuse, point, surface, subsurface) sources of a catchment or sub-catchment (for simplicity, referred to only as ‘catchment’ in this sub-section), and along the pathways of waterborne pollutant transport from that catchment to the coast, may be conceptualized and quantified as:

1 $$ r_{\text{SC}} = \frac{{M_{\text{in}} - M_{\text{out}} }}{{M_{\text{in}} }} $$

For each catchment, the total input of pollutant mass *M*_in_ is the sum of the individual pollutant inputs *m*_in,*i*_ from all *N* number of sources *i* in the catchment, and the total coastal output of pollutant mass *M*_out_ is the sum of all individual outputs *m*_out,*i*_ that reach the coast from pollutant source *i* after retention in the landscape (Fig. 1). The total and individual (source-specific) pollutant inputs to, and coastal outputs from, a catchment are thus related to each other and may be expressed as follows:

2 $$ M_{\text{in}} = \sum\limits_{i}^{N} {m_{{{\text{in,}}i}} } ;\quad M_{\text{out}} = \sum\limits_{i}^{N} {m_{{{\text{out,}}i}} } = \sum\limits_{i}^{N} {m_{{{\text{in,}}i}} \gamma_{i} \left( {L_{i} ;\;\underline{{w_{i} }} ;\;\underline{{l_{i} }};\; \underline{{p_{i} }} } \right)}, $$

where *γ*_*i*_ is the fraction of the individual pollutant input from each source *i* that ends up being transported by water to the coast along a source-specific transport pathway of length *L*_*i*_. The delivered pollutant fraction *γ*_*i*_ relates to relative pollutant retention*, r*_*i*_, along each transport pathway to the coast as *γ*_*i*_ = 1 − *r*_*i*_, with *r*_*i*_ depending on transport length *L*_*i*_ along with a number of other parameters, of which those in parameter sets $\underline{{w_{i} }}$ and $\underline{{l_{i} }}$ apply to retention that occurs in wetlands and in major lakes, respectively, and $\underline{{p_{i} }}$ is the parameter set of all other physical features and biogeochemical processes that may affect *r*_*i*_ along the pollutant transport pathways from different sources.

The parameter sets in Eq. (2) are expressed in this way in order to distinguish the retention effects of wetlands (acting through parameter set $\underline{{w_{i} }}$) in comparison with those of major lakes (set $\underline{{l_{i} }}$) and other typical landscape features (set $\underline{{p_{i} }}$) along the waterborne transport pathways (of different lengths *L*_*i*_) to the coast. Inserting Eq. (2) in Eq. (1), the relative retention *r*_SC_ for all source inputs in a catchment can be expressed as follows:

3 $$ r_{\text{SC}} = \frac{{\sum\nolimits_{i}^{{N_{i} }} {m_{{{\text{in,}}i}} \left( {1 - \gamma_{i} \left( {L_{i}; \;\underline{{w_{i} }} ;\;\underline{{l_{i} }} ;\;\underline{{p_{i} }} } \right)} \right)} }}{{M_{\text{in}} }} $$

For each type of landscape feature (wetlands, major lakes, and other features along *L*), a feature-specific relative retention (denoted *r*_*w*_, *r*_*l*_, *r*_*o*_, respectively) may also be quantified as follows:

4 $$ \begin{aligned} r_{w} &= \frac{{\sum\nolimits_{iw}^{{N_{w} }} {m_{{{\text{in,}}iw}} \left( {1 - \gamma_{iw} \left( {\underline{{w_{iw} }} } \right)} \right)} }}{{\sum\nolimits_{iw}^{{N_{w} }} {m_{{{\text{in,}}iw}} } }};\\ r_{l} & = \frac{{\sum\nolimits_{il}^{{N_{l} }} {m_{{{\text{in,}}il}} \left( {1 - \gamma_{il} \left( {\underline{{l_{il} }} } \right)} \right)} }}{{\sum\nolimits_{il}^{{N_{l} }} {m_{{{\text{in,}}il}} } }};\\ r_{o} & = \frac{{\sum\nolimits_{io}^{{N_{o} }} {m_{{{\text{in,}}io}} \left( {1 - \gamma_{io} \left( {L_{io} ;\underline{{p_{io} }} } \right)} \right)} }}{{\sum\nolimits_{io}^{{N_{o} }} {m_{{{\text{in,}}io}} } }} \\ N & = N_{w} + N_{l} + N_{o} \\ \end{aligned}, $$

where indices *N*_*w*_, *N*_*l*_, and *N*_*o*_ are the total numbers of individual source inputs *m*_*iw*_, *m*_*il*_, and *m*_*io*_ that are primarily retained by wetlands, major lakes, and other landscape features, respectively. Furthermore, for direct comparison with each feature-specific relative retention *r*_*w*_, *r*_*l*_, and *r*_*o*_ in Eq. (4), corresponding contributions *r*_SC−*w*_, *r*_SC−*l*_, and *r*_SC−*o*_ of each type of landscape feature to the total pollutant retention *r*_SC_ in a catchment can also be expressed and quantified by expanding Eq. (3) as follows:

5 $$ \begin{aligned} r_{\text{SC}} = & \,\frac{{\sum\nolimits_{iw}^{{N_{w} }} {m_{{{\text{in,}}iw}} \left( {1 - \gamma_{iw} } \right)} + \sum\nolimits_{il}^{{N_{l} }} {m_{{{\text{in,}}il}} \left( {1 - \gamma_{il} } \right)} + \sum\nolimits_{io}^{{N_{o} }} {m_{{{\text{in,}}io}} \left( {1 - \gamma_{io} } \right)} }}{{M_{\text{in}} }} \\ = & \,\frac{{r_{w} \sum\nolimits_{iw}^{{N_{w} }} {m_{{{\text{in,}}iw}} } }}{{M_{\text{in}} }} + \frac{{r_{l} \sum\nolimits_{il}^{{N_{l} }} {m_{{{\text{in,}}il}} } }}{{M_{\text{in}} }} + \frac{{r_{o} \sum\nolimits_{io}^{{N_{o} }} {m_{{{\text{in,}}io}} } }}{{M_{\text{in}} }} = r_{{{\text{SC}} - w}} + r_{{{\text{SC}} - l}} + r_{{{\text{SC}} - o}} \\ \end{aligned} $$

Comparison between Eqs. (4) and (5) shows that for any absolute pollutant retention $r_{x} \sum\nolimits_{x}^{{N_{x} }} {m_{{{\text{in,}}x}} }$ that is provided by a certain type of landscape feature (wetlands: *x* = *w*, lakes: *x* = *l*, other features: *x* = *o*) along the transport distance *L*, the relationship between that feature’s contribution *r*_SC−*x*_ to total retention *r*_SC_, and its feature-specific retention *r*_*x*_ depends on the ratio $\left( {\sum\nolimits_{x}^{{N_{x} }} {m_{{{\text{in,}}x}} } } \right)\Big/M_{\text{in}}$. With $M_{\text{in}} = \sum\nolimits_{i}^{n} {m_{{{\text{in,}}i}} }$ (Eq. 2), this ratio depends in turn on the fraction *N*_*x*_ */N* of pollutant inputs retained by landscape feature *x* relative to all pollutant inputs in a catchment. In other words, even if a landscape feature is locally very efficient in retaining pollutants transported through it, so that it has high feature-specific retention *r*_*x*_, it can only contribute significantly to the total landscape-scale retention *r*_SC_ if the fraction *N*_*x*_ */N* is large—that is if a large fraction of the total waterborne pollutant transport through the landscape goes through that feature. This is a general condition which needs to be met if any type of landscape feature is to significantly affect landscape-scale pollutant retention and thereby significantly reduce total pollutant loads through and from one or many catchments in the landscape.

By means of the above conceptualization, the main questions addressed by this study can be quantitatively stated as follows: how large is the wetland contribution *r*_SC−*w*_ in relation to (i) the total landscape retention (*r*_SC_) and (ii) the contributions of major lakes (*r*_SC−*l*_) and other typical landscape features prevailing along the hydrological transport distance (*L*) to the coast (*r*_SC−*o*_)? Regarding *r*_SC−*o*_, a subsequent question is how large is the retention contribution of the transport length *L* itself? That is, to what degree does this relatively simple-to-assess, hydro-topographically given parameter *L* determine the retention contribution *r*_SC−*o*_ of landscape features other than wetlands and major lakes? As follows, we outline a statistical analysis to investigate and quantify answers to these questions for two specific regions.

## Materials and methods

**Fig. 2:**
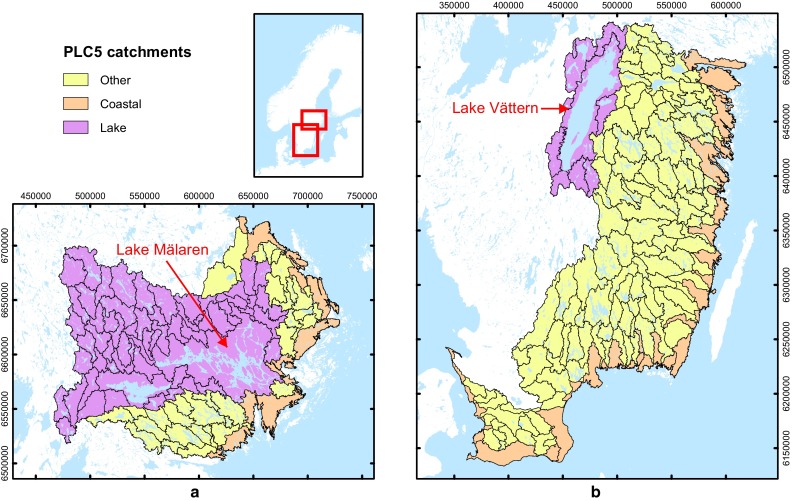
PLC5 catchments (outlined in black) in **a** the North Baltic Water Management District (WMD) and **b** the South Baltic WMD. PLC5 catchments with surface water flow and nutrient transport pathways that go through a major lake en route to their coastal outlet are marked in *purple,* while all other catchments are marked in *yellow*. The major lakes, Mälaren in the North Baltic WMD and Vättern in the South Baltic WMD, are shown

In order to quantify and compare the contributions of different landscape features (wetlands *r*_SC−*w*_, major lakes *r*_SC−*l*_ and others *r*_SC−*o*_) to total nutrient retention (*r*_SC_), we have carried out a statistical analysis. Official data on nutrient inputs (*M*_in_) and coastal outputs (*M*_out_), and on wetlands, major lakes and other landscape and catchment characteristics, were compiled separately for numerous sub-catchments within two Swedish WMDs: the North and the South Baltic (Fig. 2).

### Nutrient data

Nutrient data (nitrogen and phosphorus) were obtained from the Fifth Baltic Sea Pollutant Load Compilation (PLC5), used for national and international reporting and carried out under the auspices of Swedish Environmental Emissions Data (SMED) consortium (Swedish Environmental Protection Agency 2009a). The official PLC5 data are comprised of modeled data, based on the HBV and HBV-NP models of the Swedish Meteorological and Hydrological Institute (Lindström et al. 1997; Andersson et al. 2005). The PLC5 data are provided for sub-catchments within the WMDs (colored areas, Fig. 2). (From here on, these sub-catchments are referred to as PLC5 catchments, in accordance with the original report: Swedish Environmental Protection Agency, 2009a). Average annual nutrient inputs (*M*_in_) to PLC5 catchments are calculated for point and diffuse inputs. Point source inputs are estimated based on emission measurements or standard emission values. Diffuse source inputs are calculated using land-use leaching coefficients and runoff by means of the HBV model (Lindström et al. 1997). Atmospheric deposition rates are also included. Waterborne transport, retention processes, and the coastal output (*M*_out_) of nitrogen and phosphorus are modeled using the HBV-NP model (Andersson et al. 2005), based on the nutrient inputs (*M*_in_) and the long-term average annual run-off. The latter is calculated using measured flow data. For monitored catchments, measured nutrient data are used to calibrate the model. For unmonitored catchments, general model parameter sets are derived on a regional basis, using nutrient data available within the region. (Note that the PLC5 database does not provide information enabling measured data to be distinguished from modeled data—a fact we consider later in the discussion). Further details on the methods used by the PLC5 are available from the Swedish Environmental Protection Agency (2009a).

For each PLC5 catchment (colored areas, Fig. 2), the official data on average annual inputs (*M*_in_) and, also, associated average annual coastal outputs (*M*_out_) were available for total nitrogen (TN) and total phosphorus (TP). Here, TN and TP refer to the sum of all chemical species of nitrogen and phosphorus emanating from all pollutant sources (e.g., municipal wastewater treatment plants, agricultural sources, etc.). Also, data were available for annual inputs and coastal outputs differentiated by type of pollutant source. (Note, however, that data were not available for TN and TP differentiated by chemical species). Given *M*_in_ and *M*_out_, we quantified the relative total landscape-scale retention (*r*_SC_) for each PLC5 catchment according to Eq. (1).

### Wetlands

The retention contribution of wetlands (*r*_SC−*w*_) was investigated and quantified in terms of the correlation of total retention (*r*_SC_) to (i) the relative wetland area within each PLC5 catchment and (ii) the relative wetland area along the transport pathway from each PLC5 catchment outlet to the coastal outlet. For (i), the relative wetland area is the area of wetlands within a PLC5 catchment divided by the area of that catchment. For (ii), the relative wetland area is the sum of the area of wetlands in all downstream PLC5 sub-catchments divided by the transport distance (*L*) from each PLC5 catchment outlet to its coastal outlet. In other words, it is the wetland area per transport distance from each catchment outlet to the coastal outlet. For this latter analysis, wetland areas were obtained for all downstream PLC5 sub-catchments (Swedish Environmental Protection Agency 2009a), which are further sub-divisions of the main PLC5 catchments (Fig. 2). This further downscaling was done in order to avoid including wetlands not lying along the relevant surface flow and transport pathways to the coast. The transport distance was obtained as described further below.

For the analyses of the retention contribution of wetlands, data were obtained from the Swedish Land Cover Data (SMD), a primarily satellite-based (Landsat TM), GIS dataset with a 25 × 25 m resolution provided by Sweden’s national mapping authority (Lantmäteriet 2005). SMD maps wetlands with an area of 1 ha or greater, categorized into three main types: limnogenous marshes, peatbogs, and salt marshes. Types of wetlands included in our study were limnogenous marshes and salt marshes (Lantmäteriet 2005). These wetland types were chosen in order to ensure a physically plausible argument for wetland retention effects that may occur; otherwise, inclusion of relatively irrelevant wetland types may have hidden any statistically detectable effects. Specifically, limnogenous and salt marshes are highly relevant because they are located along the pathways of surface and subsurface flow and thereby of main waterborne nutrient transport from various sources in the landscape toward the coast. On the other hand, peatbogs are less relevant because they are water recharge zones from which some flow paths originate without having first passed through any upstream sources. However, for comparison, complementary analyses were carried out which also included peatbogs, in addition to the limnogenous and salt marshes. Furthermore, the possible effect of using an alternative wetlands database, specifically the Swedish National Wetland Inventory (VMI) (Swedish Environmental Protection Agency 2009b), which includes all three types of wetlands, was also analyzed. Moreover, the VMI database was used for an additional complementary analysis of the effect of wetland number (instead of relative wetland area) within each PLC5 catchment.

### Major lakes

In order to compare the nutrient retention contribution of wetlands (*r*_SC−*w*_)—and, separately, other landscape features (*r*_SC−*o*_)—with the contribution of major lakes (*r*_SC−*l*_), the catchments were sorted into two categories: (i) catchments with stream pathways which go through major lakes (purple, Fig. 2) and (ii) all other catchments (yellow, Fig. 2). The major lake in the North Baltic WMD is Lake Mälaren (area 1072 km^2^, or about 2.9 % of the total land area of the North Baltic WMD), which drains directly into the Stockholm archipelago—part of the Baltic Sea (Fig. 2a). The major lake in the South Baltic WMD is Lake Vättern (area 1877 km^2^, or about 3.5 % of the total land area of the South Baltic WMD), whose outlet lies nearly 100 km upstream from the Baltic Sea coast (Fig. 2b). In both the North and the South WMDs approximately 9.5 % of the total land area consists of lakes.

### Transport distance

The contribution to total *r*_SC_ of the transport distance (*L*) to the coast was quantified in terms of the correlation of total *r*_SC_ to *L* for all PLC5 catchments. The *L* values are the distance from the outlet of each PLC5 catchment to the coastal outlet along the main stream pathway. The *L* values used are thus somewhat smaller than the actual distance of waterborne nutrient transport from various sources within each PLC5 catchment to the sea. Nevertheless, *L* is still representative of the major part of the average transport distance from source to coast and, most importantly, captures the main differences in average transport distance from source to coast among the various PLC5 catchments. The *L* values were obtained using a flow network database (Swedish Hydrological and Meteorological Institute 2011), which maps surface water stream pathways from catchments to the coast via rivers, lakes, and other types of surface water bodies.

## Results

**Fig. 3:**
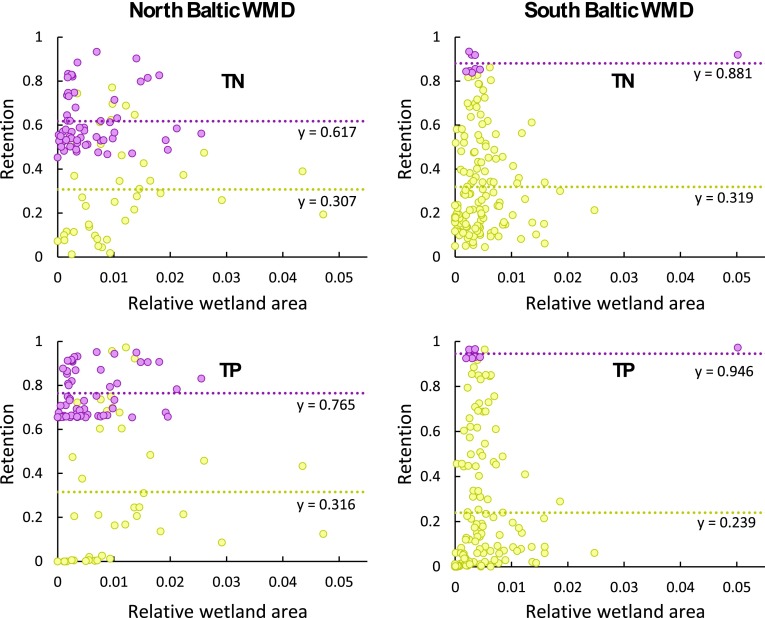
Relative nutrient retention (*r*_SC_) versus relative wetland area in the PLC5 catchments (shown in Fig. 2) for the North Baltic WMD (*left*) and the South Baltic WMD (*right*). PLC5 catchments with surface water flow and nutrient transport pathways that go through a major lake en route to their coastal outlet are marked in *purple*, while other catchments are marked in *yellow*. For both of these groups, the average relative retention for total nitrogen (TN) and total phosphorus (TP) is given, shown by the dotted lines. Linear regression over all data points yields *R* ^2^ values of 0.014 and 0.022 for TN and TP, respectively, in the North Baltic WMD, and *R* ^2^ values of 0.01 and 0.015 for TN and TP, respectively, in the South Baltic WMD

On average across all PLC5 catchments in the North and South Baltic WMDs, and for the range of 0–5 % wetland area that exists within them, there is no correlation and thus no detectable landscape-scale contribution of wetlands to the total retention of either TN or TP (Fig. 3). This can be concluded since the differences in relative wetland area do not imply any corresponding differences in total nutrient retention among the catchments. With regard to the major lakes (Mälaren and Vättern), however, their retention contribution is large for the catchments with main surface water flow and transport pathways through them (purple, Fig. 3). This can be concluded, as their average *r*_SC_ is 0.62 for TN and 0.76 for TP for the North Baltic WMD and 0.88 for TN and 0.95 for TP for the South Baltic WMD, whereas corresponding *r*_SC_ values for the other catchments are only 0.31 and 0.32, respectively, for the North Baltic WMD and 0.32 and 0.24, respectively, for the South Baltic WMD (Fig. 3). Lake Mälaren in the North Baltic WMD increases thus on average the total landscape-scale retention by a factor 2 for TN and 2.4 for TP, whereas the corresponding factors for Lake Vättern in the South Baltic WMD are somewhat greater, increasing TN and TP retention by 2.8 and 4, respectively.

**Fig. 4:**
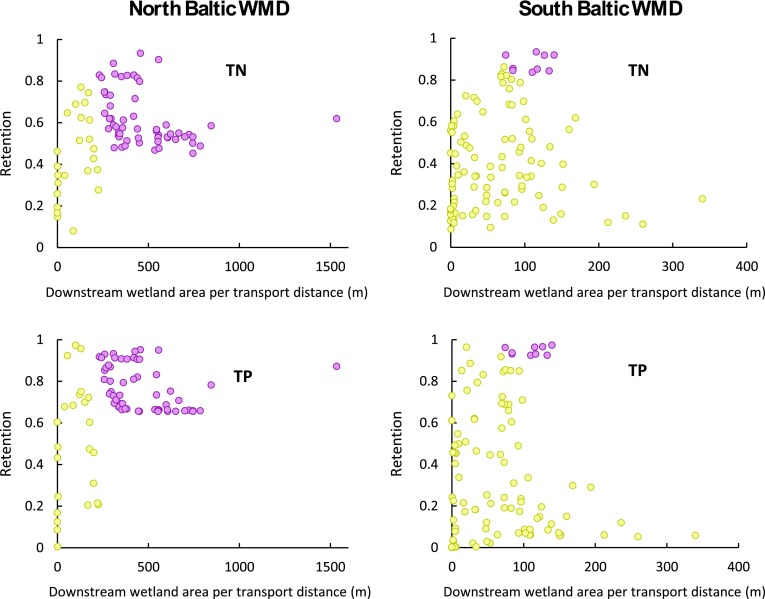
Relative nutrient retention (*r*_SC_) for total nitrogen (TN) and total phosphorus (TP) versus downstream wetland area normalized with the waterborne transport distance from each PLC5 catchment outlet to the outlet at the sea. PLC5 catchments are shown for the North Baltic WMD (*left*) and the South Baltic WMD (*right*). PLC5 catchments with surface water flow and nutrient transport pathways that go through a major lake en route to their coastal outlet are marked in *purple,* while other catchments are marked in *yellow*. Linear regression over all data points yields *R* ^2^ values of 0.053 and 0.113 for TN and TP, respectively, in the North Baltic WMD, and *R* ^2^ values of 0.007 and 0.001 for TN and TP, respectively, in the South Baltic WMD

**Fig. 5:**
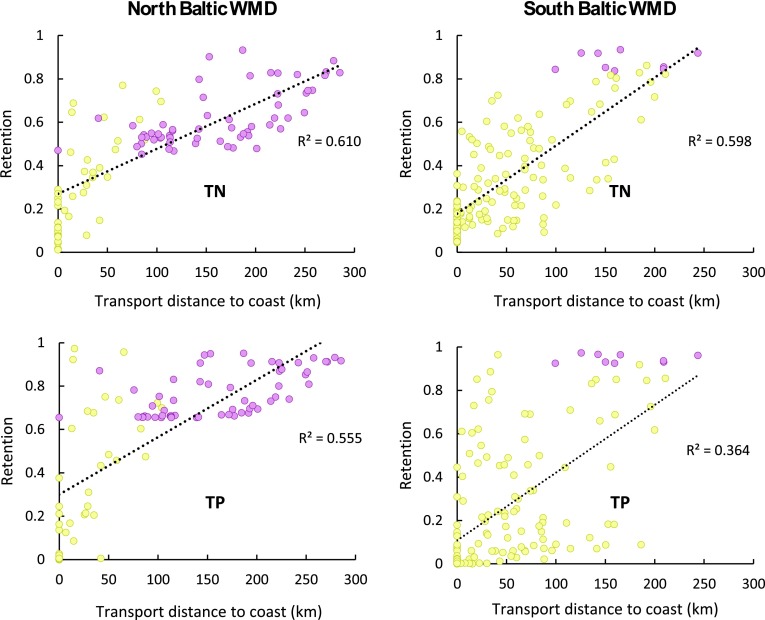
Relative nutrient retention (*r*_SC_) for total nitrogen (TN) and total phosphorus (TP) versus transport distance to the coast (km) for the PLC5 catchments (shown in Fig. 2) in the North Baltic WMD (*left*) and the South Baltic WMD (*right*). PLC5 catchments with surface water flow and nutrient transport pathways that go through a major lake en route to their coastal outlet are marked in *purple*, while other catchments are marked in *yellow*. Linear regression over all data points in each plot yields *R* ^2^ values of 0.610 and 0.555 for TN and TP, respectively, in the North Baltic WMD, and *R* ^2^ values of 0.598 and 0.364 for TN and TP, respectively, in the South Baltic WMD

The retention contribution of wetlands located downstream of each PLC5 catchment outlet, within the smaller PLC5 sub-catchments lying along the transport pathways and whose area is normalized by the transport distance *L* to the coast, is also undetectable for the range of average cross-sectional wetland width of 0–1500 m that exists downstream of the PLC5 catchments (Fig. 4). However, the total nutrient retention does show correlation with the transport distance to the sea, *L*, itself (Fig. 5). In all but one case, more than 50 % of the variation of *r*_SC_ for TN and TP in the different catchments is explicable by the simple *L* variable. For the exception—TP retention in the South Baltic WMD—the result is that 36 % of the total retention is explicable by *L*.

**Fig. 6:**
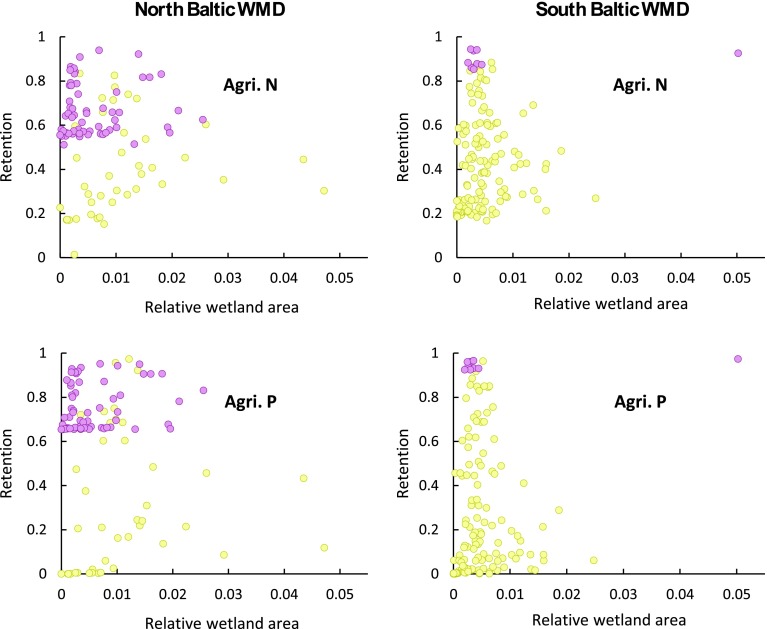
Relative nutrient retention (*r*_SC_) for all agricultural sources of nitrogen (Agri. N) and phosphorus (Agri. P) versus relative wetland area in PLC5 catchments (shown in Fig. 2) in the North Baltic WMD (*left*) and the South Baltic WMD (*right*). PLC5 catchments with surface water flow and nutrient transport pathways that go through a major lake en route to their coastal outlet are marked in purple while other catchments are marked in yellow. Linear regression over all data points yields *R* ^2^ values of 0.010 and 0.023 for Agri. N and Agri. P, respectively, in the North Baltic WMD, and *R* ^2^ values of 0.023 and 0.015 for Agri. N and Agri. P, respectively, in the South Baltic WMD

Various complementary analyses were carried out for separate pollutant source types, including diffuse sources, agricultural sources (the dominant diffuse source), and point sources. Here, we show the results for the contribution of wetlands to nutrient retention when considering only agricultural sources. Similar to the results for the analyses already presented, there is no detectable effect of wetlands on the landscape-scale retention of nutrients from agricultural sources (Fig. 6). The other complementary analyses are presented in the Electronic Supplementary Material and include retention versus relative wetland area calculated with the inclusion of peatbogs and, also, the use of another wetland database for nitrogen (Fig. S1) and for phosphorus (Fig. S2); relative nutrient retention versus the number of wetlands per PLC5 catchment (Fig. S3); relative nutrient retention versus wetland area for diffuse sources (Fig. S4); and relative nutrient retention versus wetland area for point sources (Fig. S5). None of these complementary analyses revealed any correlation between retention and wetland characteristics at a landscape-scale; thus, the overall result of an undetectable effect of wetlands at landscape-scale remains unchanged.

## Discussion

The contribution of wetlands to the regulating ecosystem service of nutrient retention at the landscape-scale is undetectable in both the North and South Baltic WMDs. This is explained by the condition, derived from the general theoretical model. This condition clarifies that for any type of landscape feature to contribute significantly to the regulating ecosystem service of pollutant retention at landscape-scale, a large fraction of the total waterborne pollutant transport through the landscape must pass through that feature type. This condition is indeed fulfilled for the basic landscape feature of waterborne transport distance from catchment to coast, which is non-zero for all but the smallest sub-catchments right at the coast. Previous research has shown that, for any given average rate of retention that may prevail in a landscape, the resulting pollutant retention depends on the combination (product) of that retention rate and the average time of transport through the landscape, with the latter greatly dependent on transport distance (Persson and Destouni 2009, 2011; Destouni et al. 2010; Cvetkovic et al. 2012). The transport distance to the sea has, here, been found to account for much of the variation in nutrient retention (61 and 60 % for TN and 56 and 36 % for TP in the North and South Baltic WMDs, respectively), which is fully consistent with the previous research. The condition is also fulfilled for all sub-catchments with major lakes downstream, through which most of the water flow and waterborne pollutant transport to the coast takes place. In contrast, this condition is typically not fulfilled for the large-scale characteristics of wetlands in the landscapes analyzed here—including where the wetland area relative to the sub-catchment area is up to 5 % in total.

Regarding the statistical analysis of the landscape-scale contribution of wetlands to retention, could any potential errors in the data sources used have affected the result? For the wetlands data, the result was consistent for both the landcov er dataset, SMD, and the wetland inventory, VMI. Thus, potential errors in either of these datasets, regarding the area or type of wetland, are unlikely to change the statistical result. For the official PLC5 data (Swedish Environmental Protection Agency 2009a), the modeled data for each PLC5 catchment are either calibrated to systematically monitored data or are based on region-specific parameters. A pertinent question is whether or not catchments with data modeled using region-specific parameters accurately capture wetland retention effects at the landscape scale. In Sweden—and in other countries—environmental managers use such modeled data to appreciate and officially report nutrient loads. They also use such data when making decisions on mitigating nutrient loads. These datasets are subject to errors. Nevertheless, despite dataset errors, if wetlands are to be objectively considered as large-scale mitigation measures, then their nutrient retention effects should be detectable. Indeed, if this modeling subsumes wetland retention effects into other landscape features, the present results would be pertinent in that they indicate that such a model bias possibly exists. However, given calibration and consistency with observed data in monitored catchments and adequate modeling in unmonitored catchments, one may expect that the model sufficiently represents retention processes.

It should be emphasized that the results of this study do not contradict previous research which shows that wetlands may be locally efficient at retaining nutrients (Reddy et al. 1999; Saunders and Kalff 2001; Mitsch et al. 2005; Vymazal 2007) as well as other types of pollutants (Aisling et al. 1999; ERMITE-Consortium 2004; Baresel et al. 2006). Thus, the results do not suggest that wetlands should be disregarded as appropriate local mitigation measures, as they could greatly benefit local downstream water bodies that are affected by pollution. However, as the general analytical model and the region-specific results both show, it should be recognized that many small wetlands distributed throughout the landscape, which occupy only a small fraction of a catchment, cannot have a significant effect on landscape-scale pollutant retention with regard to major downstream water bodies, such as the Baltic Sea, unless the wetlands are placed in such a way that much of the total waterborne pollutant transport to the major downstream recipient actually passes through them.

Attention to the actual pathways of waterborne pollutant transport at large catchment and landscape scales is therefore required in planning and decision-making concerned with the construction and restoration of wetlands—especially if these wetlands are to provide the desired regulating ecosystem service of reducing pollutant loads to major downstream water systems. In order to fulfil the general condition, which requires that a significant proportion of the waterborne transport to a major downstream recipient actually passes through existing or planned wetlands or wetland systems, ‘hotspots’ for wetland placement in the landscape should be identified.

An important question, even though difficult and requiring further research, is if and how such ‘hotspots’ for wetlands can be identified in order to mitigate major diffuse pollutant loads—including those from environmental-legacy, subsurface sources. These legacy sources are due to past pollutant inputs that are either slowly transported by subsurface water (soil water and groundwater) or reside in immobile zones of soils, aquifers, sediments, and engineered subsurface formations (e.g., mining or municipal waste deposits). Such legacy sources have been found to continuously release pollutants into the mobile aqueous phase of soil water and groundwater and, through these, into surface water (ERMITE-Consortium, 2004; Baresel et al., 2006; Darracq et al., 2008; Basu et al., 2010; Destouni et al., 2010). Recognition and improved knowledge of such legacy sources is crucial for water management planners in order to enable them to make better informed decisions about the effectiveness and efficiency of wetland construction, restoration, and protection programs specifically aimed at enhancing the landscape-scale ecosystem service of waterborne pollutant load mitigation.

## Conclusion

A general analytical model has been developed and a statistical analysis of region-specific data has been carried out to answer the questions pertaining to the regulating ecosystem service of pollutant retention: (i) what is the large-scale contribution of wetlands to total pollutant retention in the landscape and (ii) how does this contribution compare with that of other landscape features? As a general condition, the analytical model clarifies that a landscape feature can only contribute significantly to the total large-scale retention if a large fraction of the total waterborne pollutant transport goes through that feature. Consistent with this condition, the results of the statistical analyses reveal an undetectable effect of wetlands on nutrient retention at the landscape-scale for the catchments in the two Swedish WMDs investigated, the North and the South Baltic. Instead, two other landscape features, the distance of waterborne nutrient transport and the presence of major lakes along the pathways of that transport to the coast, explain much of the variation in the landscape-scale nutrient retention.

In general, these results emphasize the need for informed consideration of the large-scale pathway distributions of water flow and pollutant transport through catchments in order to accurately understand and quantify the large-scale ecosystem service of pollutant retention. This should better enable choice of efficient strategies and measures for restoring and protecting inland and coastal water quality. In particular, construction or restoration of wetlands for this purpose may be an inefficient use of typically limited resources for environmental management—particularly if minor wetlands with a relatively small total area are sited far upstream from major coastal-marine systems, such as the Baltic Sea. This especially applies if such wetlands are sited upstream of major lakes, which are not themselves in need of restoration or added protection.

## Electronic supplementary material

Below is the link to the electronic supplementary material.

Supplementary material 1 (PDF 237 kb)
