# Supplementary material for: Dissecting the ecosystem service of large-scale pollutant retention: The role of wetlands and other landscape features
Source: Ambio. 2015 Jan 9;44(Suppl 1):127–37. doi: 10.1007/s13280-014-0594-8 (PMC4288994; doi:10.1007/s13280-014-0594-8)
Supplement: Supplementary file 1 — Supplementary material 1 (PDF 237 kb) [file 13280_2014_594_MOESM1_ESM.pdf]

AMBIO

Electronic Supplementary Material

Dissecting the ecosystem service of large-scale nutrient retention: the role of wetlands and other landscape features

Andrew Quin, Fernando Jaramillo, Georgia Destouni

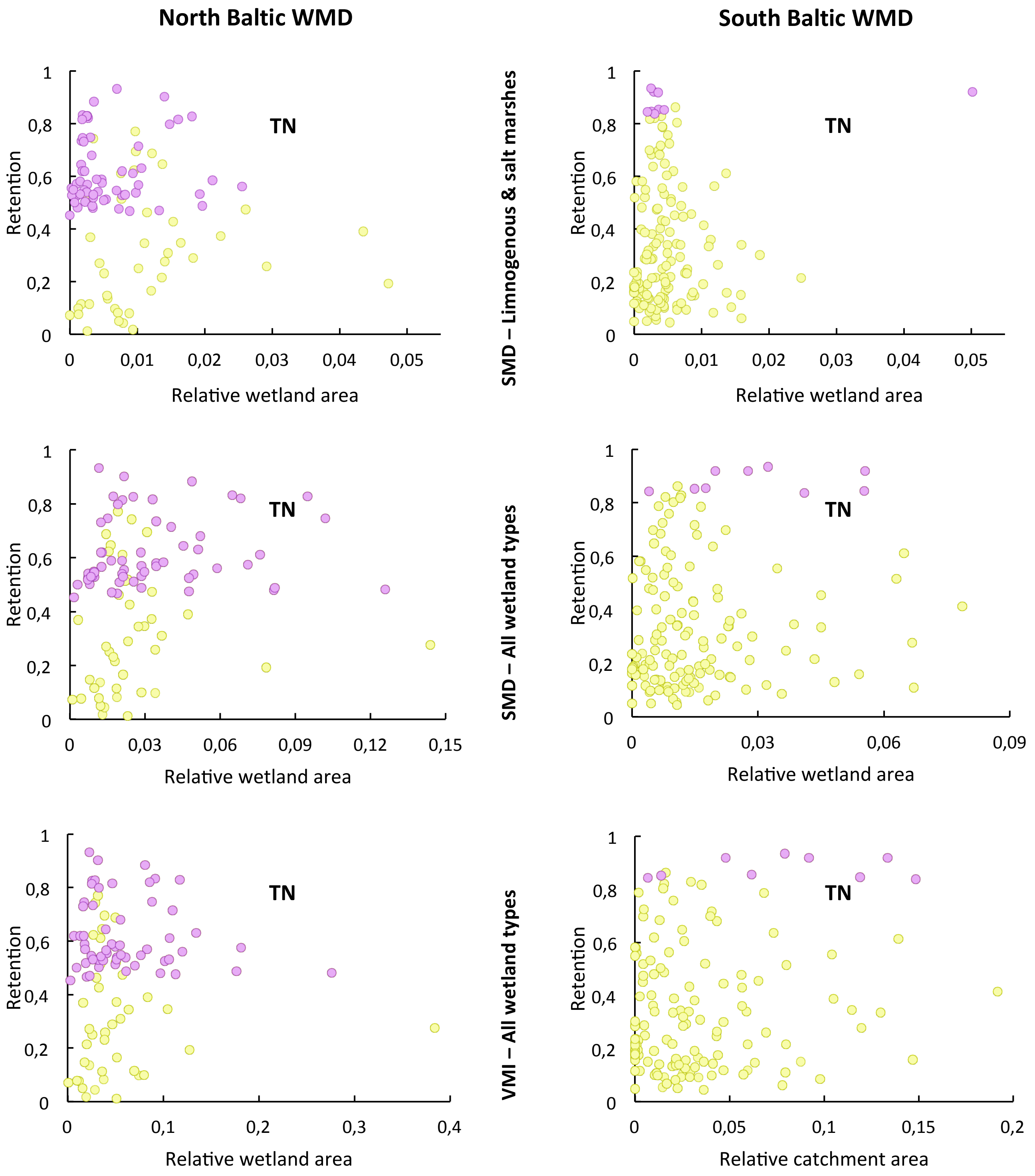

Fig. S1 Relative nutrient retention (r_{SC}) for all sources of nitrogen (TN) versus wetland area in all PLC5 catchments in the North Baltic Water Management District (WMD) (left panels) and the South Baltic WMD (right panels). PLC5 catchments with flow and nutrient transport pathways that go through a major lake en route to their coastal outlet are marked in purple while other catchments are marked in yellow. For the uppermost panels, the relative wetland area in each PLC5 catchment is calculated using the Swedish Land Cover Data (SMD) including only limnogenous marshes and salt marshes, as stated in Materials and Methods. (These panels are thus the same as the upper panels in Fig. 3 and are shown here to allow comparison with the panels below.) For the panels in the middle, the relative wetland area is calculated using SMD including all wetland types. For the panels at the bottom, the relative wetland area is calculated using another wetlands

database, the Swedish National Wetland Inventory (VMI) and also includes all wetland types. Linear regression over all data points does not show any correlation between relative retention for nitrogen and relative wetland area for these plots.

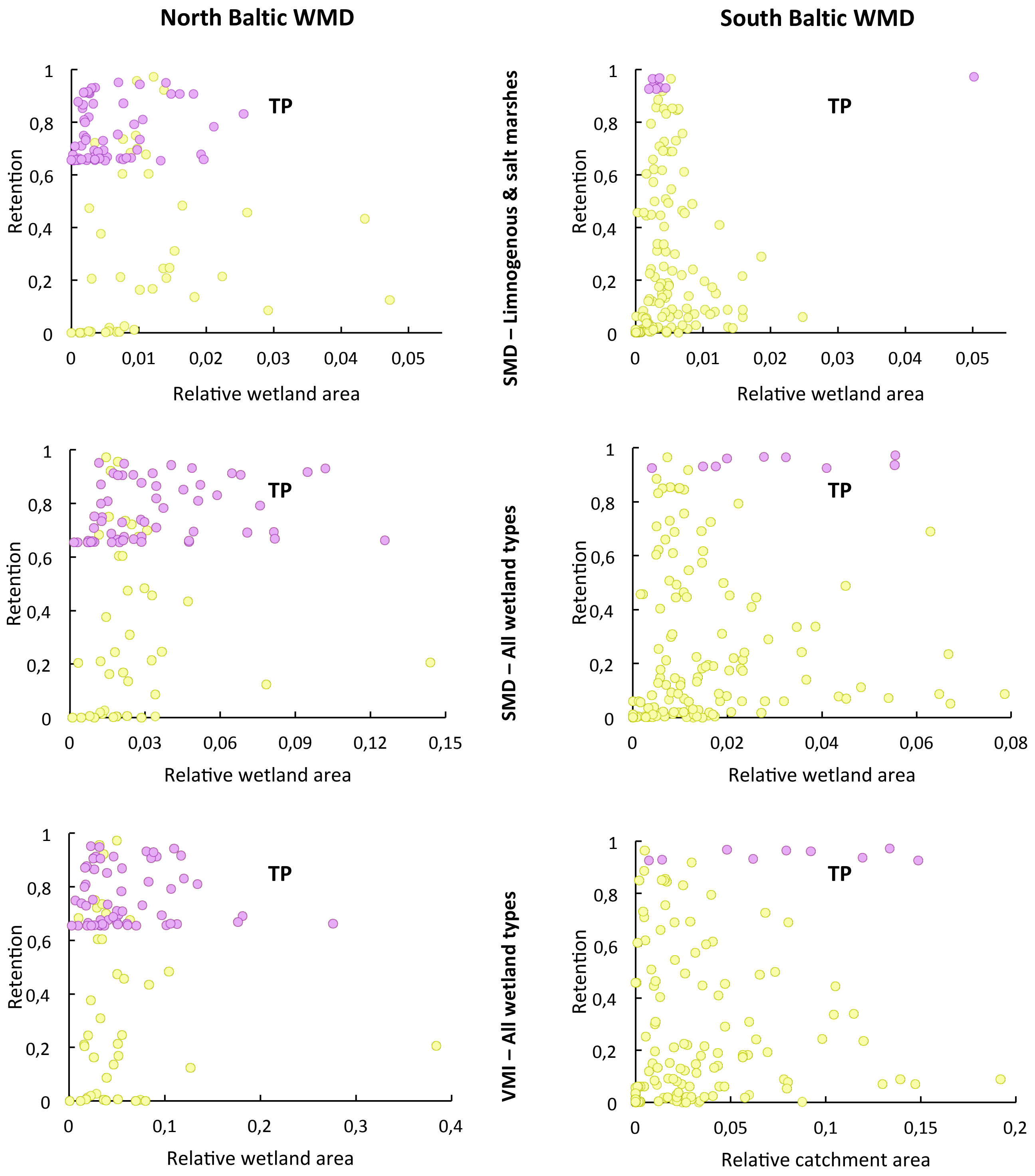

Fig. S2 Relative nutrient retention (r_{SC}) for all sources of phosphorus (TP) versus wetland area in all PLC5 catchments in the North Baltic WMD (left panels) and the South Baltic WMD (right panels). PLC5 catchments with flow and nutrient transport pathways that go through a major lake en route to their coastal outlet are marked in purple while other catchments are marked in yellow. For the uppermost panels, the relative wetland area in each PLC5 catchment is calculated using the Swedish Land Cover Data (SMD) including only limnogenous marshes and salt marshes, as stated in Materials and Methods. (These panels are thus the same as the upper panels in Fig. 3 and are shown here to allow comparison with the panels below.) For the panels in the middle, the relative wetland area is calculated using SMD including all wetland types. For the panels at the bottom, the relative wetland area is calculated using another wetlands database, the Swedish National

Wetland Inventory (VMI) and also includes all wetland types. Linear regression over all data points does not show any correlation between relative retention for phosphorus and relative wetland area for these plots.

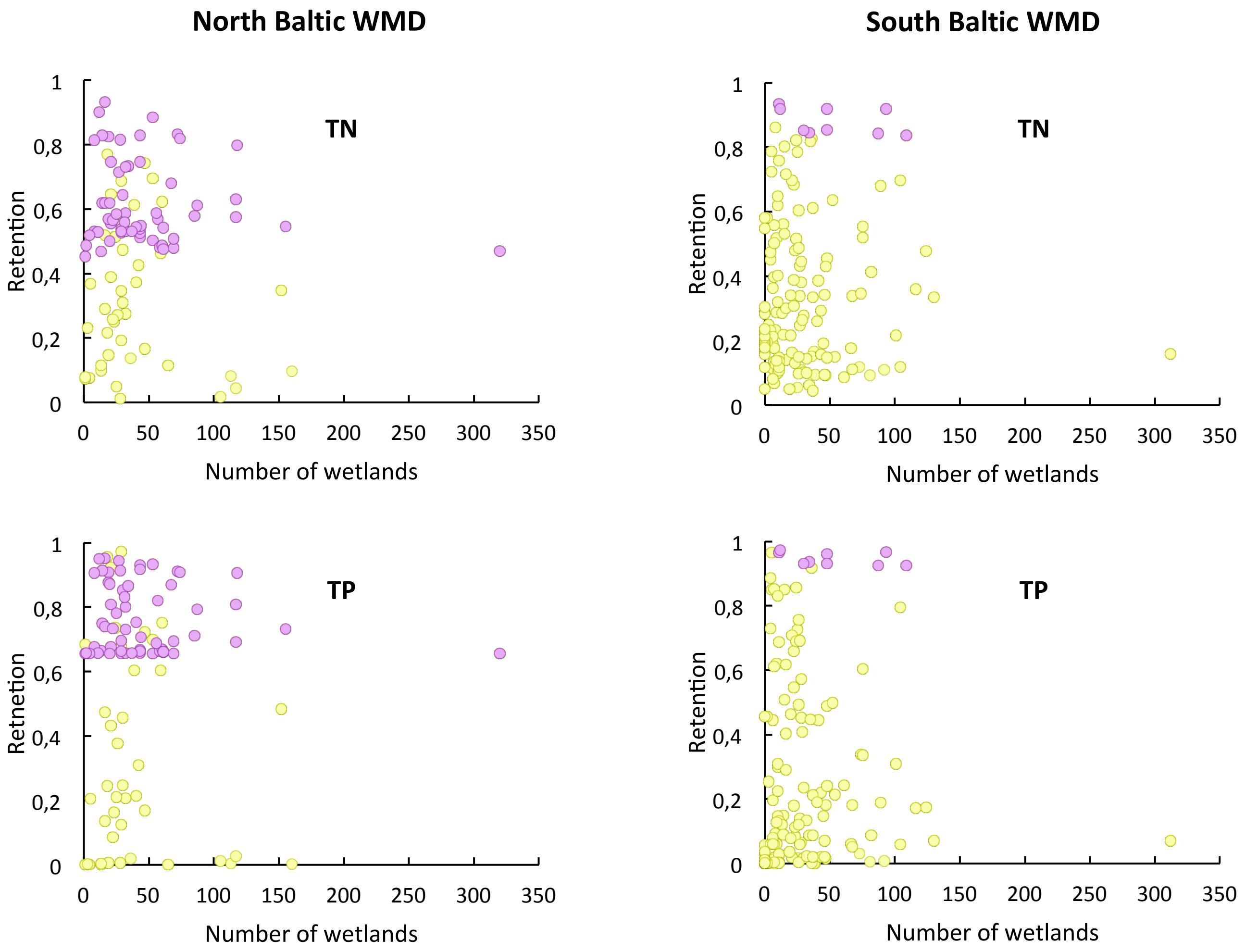

Fig. S3 Relative nutrient retention (r_{SC}) for all sources of nitrogen (TN) and phosphorus (TP) versus number of wetlands in PLC5 catchments (shown in Figure 2) in the North Baltic WMD (left panels) and the South Baltic WMD (right panels). PLC5 catchments with flow and nutrient transport pathways that go through a major lake en route to their coastal outlet are marked in purple while other catchments are marked in yellow. Linear regression over all data points yields R^2 values of 0.001 and <0.001 for TN and TP, respectively, in the North Baltic WMD, and R^2 values of 0.004 and 0.003 for TN and TP, respectively, in the South Baltic WMD.

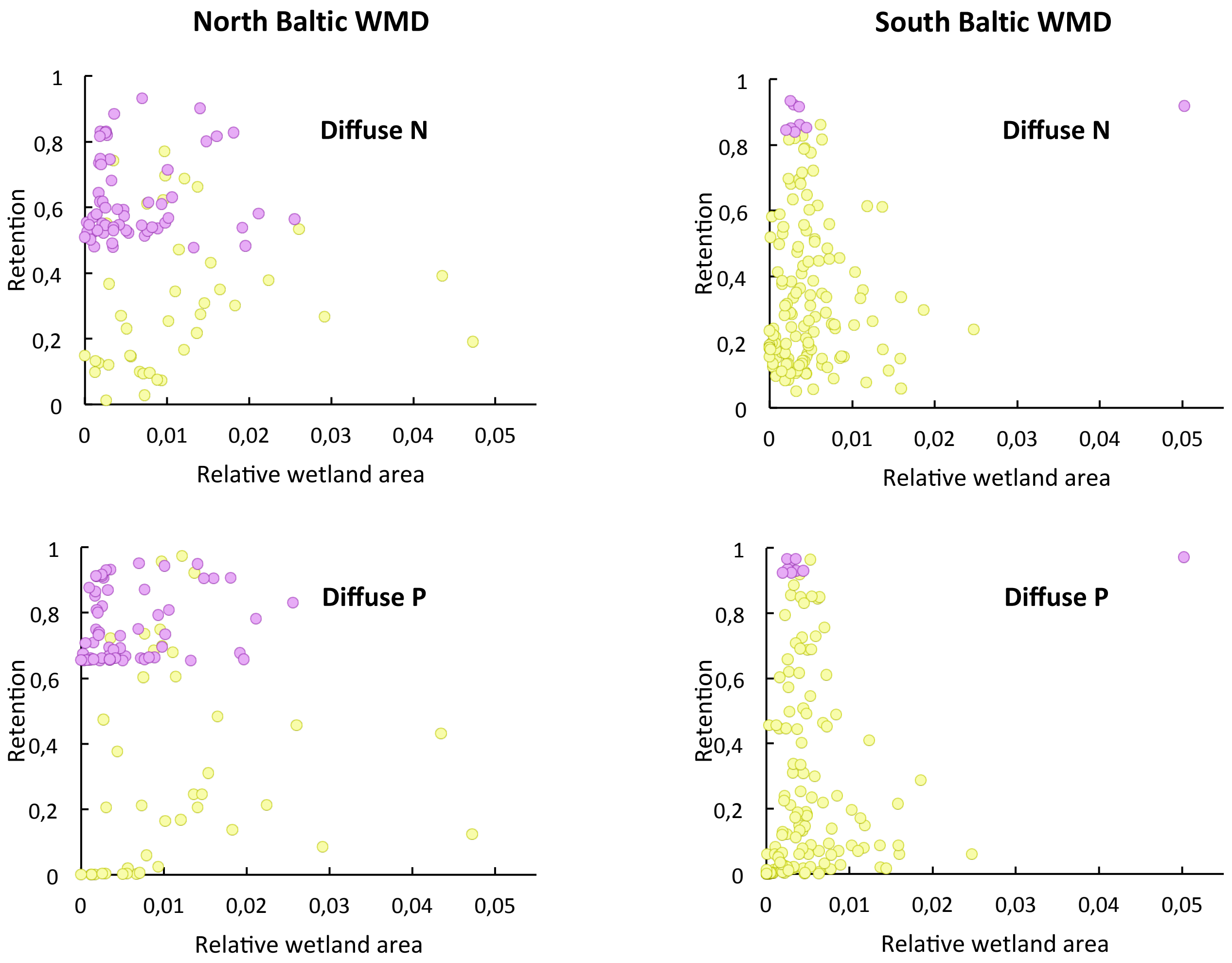

Fig. S4 Relative nutrient retention (r_{SC}) for all diffuse sources of nitrogen (Diffuse N) and phosphorus (Diffuse P) versus relative wetland area in PLC5 catchments (shown in Figure 2) in the North Baltic WMD (left panels) and the South Baltic WMD (right panels). PLC5 catchments with flow and nutrient transport pathways that go through a major lake en route to their coastal outlet are marked in purple while other catchments are marked in yellow. Linear regression over all data points yields R^2 values of 0.015 and 0.022 for Diffuse N and Diffuse P, respectively, in the North Baltic WMD, and R^2 values of 0.010 and 0.015 for Diffuse N and Diffuse P, respectively, in the South Baltic WMD.

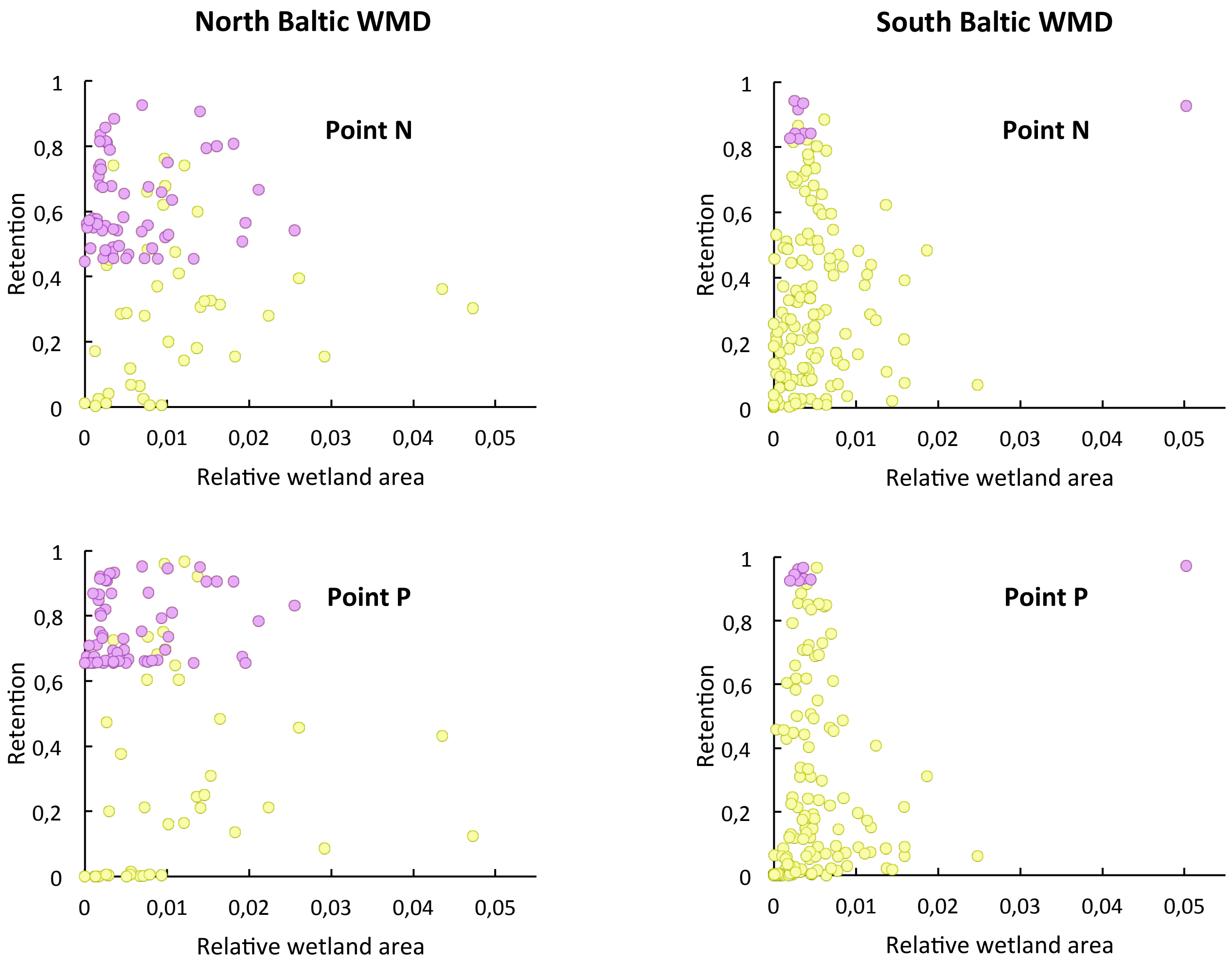

Fig. S5 Relative nutrient retention (r_{SC}) for all point sources of nitrogen (Point N) and phosphorus (Point P) versus relative wetland area in PLC5 catchments (shown in Figure 2) in the North Baltic WMD (left panels) and the South Baltic WMD (right panels). PLC5 catchments with flow and nutrient transport pathways that go through a major lake en route to their coastal outlet are marked in purple while other catchments are marked in yellow. Linear regression over all data points yields R^2 values of 0.016 and 0.022 for Point N and Point P, respectively, in the North Baltic WMD, and R^2 values of 0.019 and 0.015 for Point N and Point P, respectively, in the South Baltic WMD.
